# *Cathetus yuanjiangensis* (Phyllanthaceae, Phyllantheae), a New Species from Southwest China, with a New Combination and an Updated Key to *C.* Subgen. *Cathetus*

**DOI:** 10.3390/plants15152274

**Published:** 2026-07-25

**Authors:** Feng Yang, Chao Chen, Zhuo Li, Jian-Yong Wu, Hui Fan, Huan-Chong Wang

**Affiliations:** 1 School of Ecology and Environmental Science, Yunnan University, Kunming 650091, China; yfde828@gmail.com (F.Y.); lizhuooo1117@163.com (Z.L.); 2 Yunnan Key Laboratory of Forest Ecosystem Stability and Global Change, Xishuangbanna Tropical Botanical Garden, Chinese Academy of Sciences, Mengla 666303, China; chenchao@xtbg.ac.cn; 3 Laboratory of Tropical Forest Ecology, Xishuangbanna Tropical Botanical Garden, Chinese Academy of Sciences, Mengla 666303, China; 4 Yuanjiang Savanna Ecosystem Research Station, Xishuangbanna Tropical Botanical Garden, Chinese Academy of Sciences, Yuanjiang 653300, China; 5 Yuxi Forestry and Grassland Bureau, Yuxi 653100, China; 13987710327@163.com; 6 Yunnan Yuanjiang National Nature Reserve Management Bureau, Yuanjiang 653300, China; 13388775185@163.com; 7 State Key Laboratory of Vegetation Structure, Function and Construction (VegLab), Kunming 650091, China

**Keywords:** Phyllantheae, *Phyllanthus*, Yuanjiang dry-hot valley, plastid genome, phylogeny

## Abstract

*Cathetus yuanjiangensis* Huan C. Wang, Feng Yang et Chao Chen (Phyllantheae, Phyllanthaceae), a new species from the dry-hot valleys of Southwest China, is described and illustrated herein. Morphologically, the new species can be easily distinguished from its congeners by the following combination of characters: leaves 1.5–4.5 cm long, 1–2 cm wide; staminate flowers sepals spreading 1.5–2.0 mm long; filaments connate into a 2–3 mm long column; styles 3, ca. 1.6 mm long, connate into a ca. 0.5 mm long column at base; stigmas spreading, slender, oblong, each bifid to 1/2, the lobes linear. Phylogenetic analyses based on two nuclear (ITS and *PHYC*) and three chloroplast (*mat*K, *acc*D-*psa*I and *trn*S-*trn*G) DNA makers recover *C. yuanjiangensis* as the sister clade to *C. fasciculate* among the sampled taxa. Detailed morphological descriptions, photographs, pollen morphology, and complete plastid genome characteristics of the new species are provided, along with a distribution map and a preliminary conservation assessment. Additionally, we propose a new combination, *Cathetus beillei* (Hutch.) Huan C. Wang et Feng Yang, based on the basionym *Phyllanthus beillei* Hutch., which was originally published in Flora of Tropical Africa. An updated identification key for all recognized species of *C.* subgen. *Cathetus* is provided.

## 1. Introduction

The tribe Phyllantheae is the largest tribe in the family Phyllanthaceae, comprising more than 1200 species [1]. The systematic classification of this tribe has long been plagued by the paraphyly of *Phyllanthus* s.l. [1]. Since the genus *Phyllanthus* L. was established by Linnaeus in 1753, *Phyllanthus* has been treated as a broad group containing nearly 900 species due to convergent morphological characteristics and extremely high morphological diversity [2]. However, recent molecular studies clearly demonstrated that *Phyllanthus* (in the traditional sense) is paraphyletic since *Glochidion* J.R. Forst. et G. Forst., *Breynia* J.R. Forst. et G. Forst. and *Synostemon* F. Muell. are nested within this clade [3,4,5]. To resolve the paraphyly of *Phyllanthus* s.l., Bouman et al. [5] proposed a phylogenetic classification that divides *Phyllanthus* s.l. into several smaller, monophyletic genera that can be characterized morphologically, based on phylogenetic analyses of nuclear genes and plastid genes, altogether with morphological and palynological evidence, particularly pollen morphological characteristics. The classification system of Bouman et al. [5] has been accepted by many studies [6,7,8,9,10], while others still accept and use paraphyletic *Phyllanthus* s.l. [11].

*Cathetus* Lour. was first formally described by Loureiro in 1790 based on the type species *Cathetus fasciculata* Lour. (later synonymized with the formerly recognized *Phyllanthus cochinchinensis* Spreng.) [12]. For over a century thereafter, *Cathetus* was subsumed in the genus *Phyllanthus* s.l. [5]. Subsequent classifications [13,14,15] retained this broad circumscription, treating *Cathetus* allied taxa as subgenera or sections of *Phyllanthus* s.l. It was not until 2022 that Bouman et al. reinstated and redefined *Cathetus* as an independent genus [5]. The reinstated and redefined genus *Cathetus* includes two phylogenetically supported subgenera, namely subgen. *Cathetus* and subgen. *Macraea* (Wight) R.W. Bouman. Among them, subgen. *Cathetus* (formerly *P.* subgen. *Ceramanthus* (Hassk.) Brunel) consists of six species, characterized by the complete fusion of stamen filaments and connectives forming a staminal column, pantoporate or brevicolporate pollen grains, and a coarsely reticulate exine. Subgen. *Macraea* (formerly *P.* subgen. *Macraea* (Wight) Baillon) includes 34 species, distinguished by free or basally connate stamen filaments, poriform (peltate) pollen apertures, typically distichous leaves, and a growth habit ranging from herbs and subshrubs to trees. The newly delimited *Cathetus* has an Old World tropical and subtropical distribution, occurring in Africa, mainland Asia, the Malay Archipelago, Australia and the Pacific region.

There are five species native to China, namely *Cathetus filipes* D. X. Nong, B.Y. Huang &et Z. Xiong, *C. clarkei* (Hook.f.) R.W. Bouman, *C. fasciculata*, *C. ussuriensis* (Rupr. et Maxim.) R.W. Bouman, and *C. virgatus* (Forster) R.W. Bouman. In addition, *C. myrtifolius* (Wight) R.W. Bouman, native to Sri Lanka, has been introduced for ornamental cultivation [10,16]. *Cathetus* is also widely distributed in China, and is found in all provinces and regions except the northwest region. Nevertheless, the diversity and taxonomy of this genus in China remain poorly understood. During our recent fieldwork in China, an unknown species of *Cathetus* with large leaves was encountered in Yuanjiang Valley, Yunnan Province. After a detailed study of its morphology and analysis of relevant literature [5,16,17,18,19,20,21,22,23], we hypothesized that this plant represents a new species of *Cathetus*.

In the present study, we formally describe this plant as a new species, namely *Cathetus yuanjiangensis* Huan C. Wang, Feng Yang et Chao Chen sp. nov., providing photographs, pollen morphology, a plastid genome, a distribution map, a preliminary conservation assessment, and taxonomic notes. Phylogenetic analysis based on nuclear (ITS and *PHYC*) and chloroplast (*mat*K, *acc*D-*psa*I and *trn*S-*trn*G) sequence data was conducted to test the phylogenetic placement of *C*. *yuanjiangensis* within *Cathetus*. Additionally, we herein propose a new combination, *C. beillei* (Hutch.) Huan C. Wang et Feng Yang comb. nov., based on the basionym *Phyllanthus beillei* Hutch., which was originally published in Flora of Tropical Africa by Hutchinson in 1912. An updated identification key for all currently recognized species of the subgen. *Cathetus* is presented.

## 2. Results

### 2.1. Characteristics of the Plastid Genome, nrDNA and PHYC

**Figure A1:**
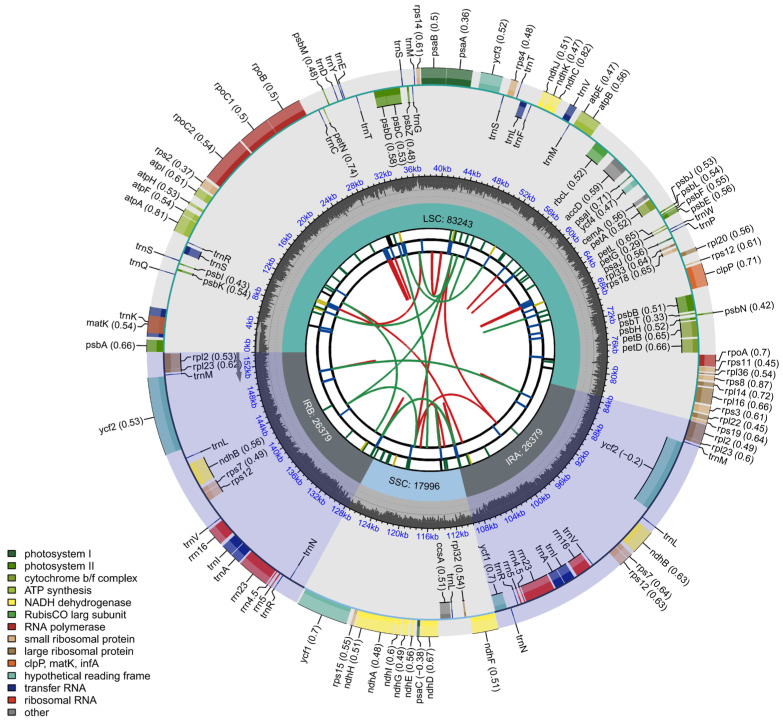
Schematic map of overall features of the plastid genome of *Cathetus yuanjiangensis*. The map contains six tracks. From the center outward, the first track shows the dispersed repeats. The dispersed repeats consist of direct (D) and Palindromic (P) repeats, connected with red and green arcs. The second track shows the long tandem repeats as short blue bars. The third track shows the short tandem repeats or microsatellite sequences as short bars with different colors. The small single-copy (SSC), inverted repeat (IRa and IRb), and large single-copy (LSC) regions are shown on the fourth track. The GC content along the genome is plotted on the fifth track. IThe genes are shown on the sixth track. The optional codon usage bias is displayed in the parenthesis after the gene name. Genes are color-coded by their functional classification. The transcription directions for the inner and outer genes are clockwise and anticlockwise, respectively. The functional classification of the genes is shown in the bottom left corner.

The complete plastid genome sequences of *Cathetus yuanjiangensis* comprised 152,879–153,997 bp. Each genome had the circular and typical quadripartite structure and contained a large single-copy region (LSC, 83,243–83,353 bp), a small single-copy region (SSC, 17,996–18,004 bp), and two inverted sequence repeat regions (IRs, 25,761–26,379 bp each) (Figure A1). The percentage of GC in the whole genome was 37.0%. We annotated a total of 128 functional genes, including 85 protein-coding genes, 36 tRNA genes, and 8 rRNA genes (Figure A1, Table A1). These plastid genomes were used as a source of *mat*K, *acc*D-*psa*I and *trn*S-*trn*G in the phylogenetic analyses. The assembled nrDNA repeats, including the ETS, 18S, ITS1, 5.8S, ITS2, and 26S regions, were 8884–8903 bp in length, with a GC content of 55.2%. All *PHYC* genes were 718–805 bp in length, with a GC content of 41.6–42.1%. The newly generated plastid genome, nrDNA and *PHYC* sequences have been deposited in GenBank (Table A2).

### 2.2. Molecular Phylogenetic Analysis

**Figure 1:**
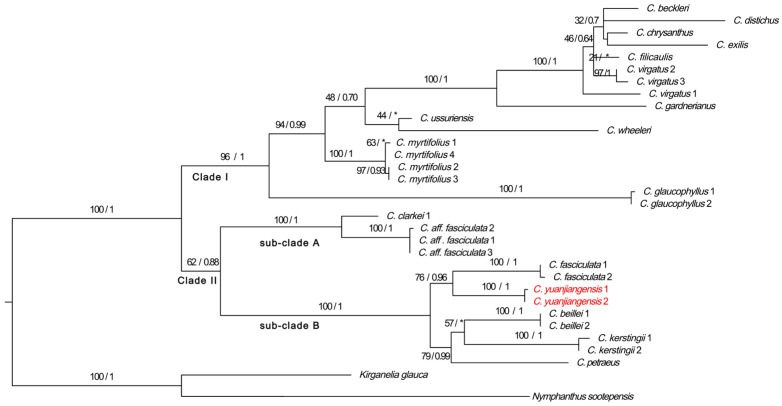
The phylogenetic position of *Cathetus yuanjiangensis* in the genus *Cathetus* based on ML and BI methods using concatenated matrix sequences from two nuclear markers, ITS and *PHYC*. The positions of *C. yuanjiangensis* are marked by red text. The asterisk (*) indicates nodes not supported in ML phylogenetic tree.

**Figure 2:**
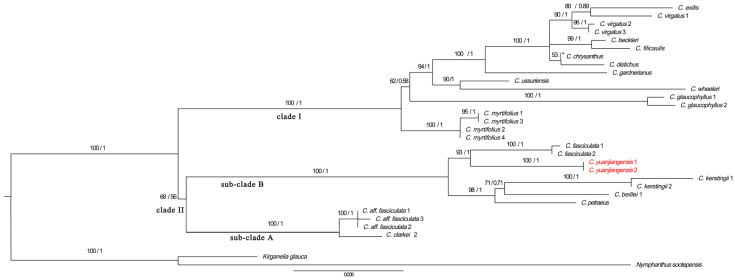
The phylogenetic position of *Cathetus yuanjiangensis* in the genus *Cathetus* based on ML and BI methods using concatenated matrix sequences from three plastid markers: *mat*K, *acc*D-*psa*I and *trn*S-*trn*G. The positions of *C. yuanjiangensis* are marked by red text. The asterisk (*) indicates nodes not supported in ML phylogenetic tree.

The alignment length of the concatenated nuclear set was 1486 bp, and the percentage of parsimony informative sites was 22.01%. The concatenated three plastid regions had a length of 2457 bp for phylogenetic analysis, with 11.19% parsimony informative sites. Similar topologies were obtained in the Maximum Likelihood (ML) and Bayesian Inference (BI) analyses using two different matrix sequences of concatenated molecular markers (Figure 1 and Figure 2).

The phylogenetic trees recovered *Cathetus* as two major highly supported lineages. Eleven sampled species from subgenus *Macraea* formed clade I. Clade II lineage could be further divided into two sub-clades. Sub-clade A included *C. clarkei* and one taxon allied to *C. fasciculata*, whereas sub-clade B consisted of an Asian clade and an African clade. Within the Asian clade, all accessions of *C. yuanjiangensis* formed a strongly supported lineage (Figure 1 and Figure 2: 100%/1) and were recovered as sister to *C. fasciculata* among the sampled taxa (Figure 1: 76%/0.96; Figure 2: 93%/1). Within the African clade, *C. beillei* was recovered as the likely sister of *C. kerstingii*, but this relationship had low support and differed between the ML and BI analyses (Figure 1: 57%/*; Figure 2: 71%/0.71). *Cathetus petraeus* occupied a more basal position as sister to the clade containing *C. beillei* and *C. kerstingii* (Figure 1: 79%/0.99; Figure 2: 98%/1).

### 2.3. Taxonomic Treatment

**Figure 3:**
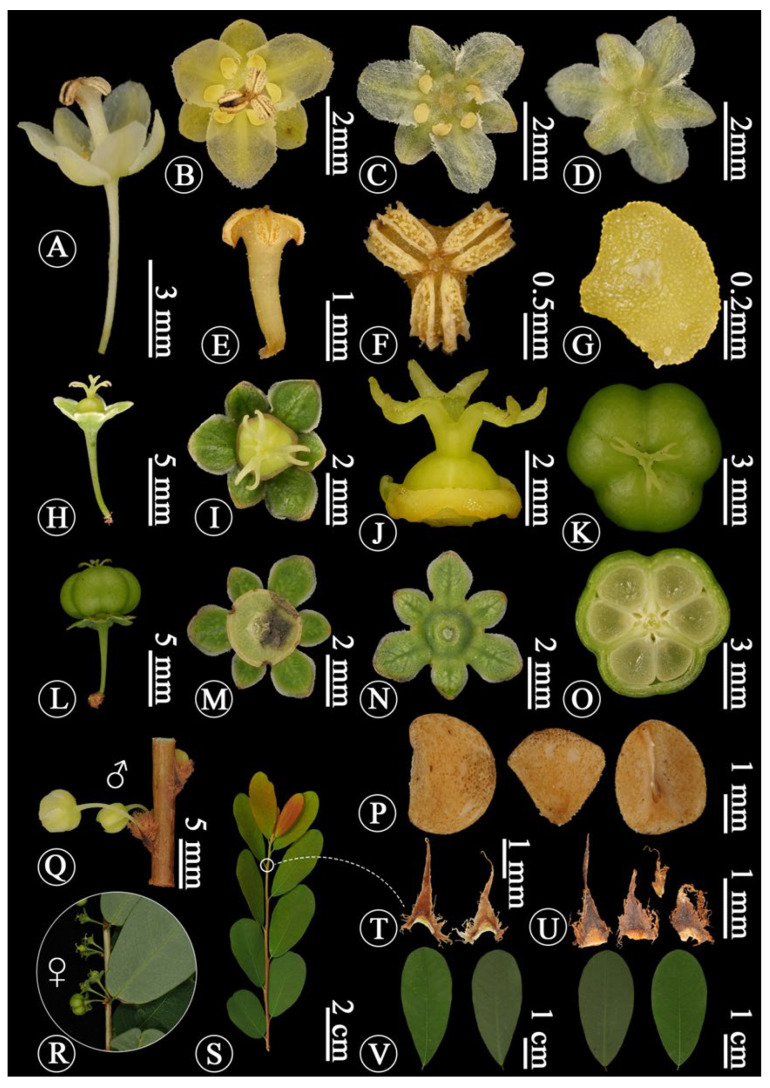
Anatomical diagram of *Cathetus yuanjiangensis* (voucher: Chao Chen YJ31054, YUKU). (**A**–**G**) Staminate flowers and related structures; (**A**) lateral view of staminate flower; (**B**) top view of staminate flower; (**C**) staminate flower with stamens removed, showing 6 disc glands, one of which has fallen off; (**D**) dorsal view of a staminate flower; (**E**) filaments connate into a column; (**F**) Dehiscing anthers; (**G**) a disc gland. (**H**–**O**) Pistillate flowers and fruits; (**H**) lateral view of pistillate flower; (**I**) top view of pistillate flower; (**J**) disc, pistil and stigma of a pistillate flower; (**K**) young fruit; (**L**) lateral view of mature fruit; (**M**) mature fruit with fruit removed, showing the persistent disc and calyx; (**N**) dorsal view of pistillate flower; (**O**) cross-section of fruit. (**P**) Seeds, showing lateral, dorsal, and ventral views (from left to right). (**Q**) Staminate flowers (♂) fasciculata in leaf axils. (**R**) Pistillate flowers (♀) fasciculata in leaf axils. (**S**) Branchlet. (**T**) Stipules. (**U**) Bracts. (**V**) Adaxial and abaxial surfaces of leaves.

**Figure 4:**
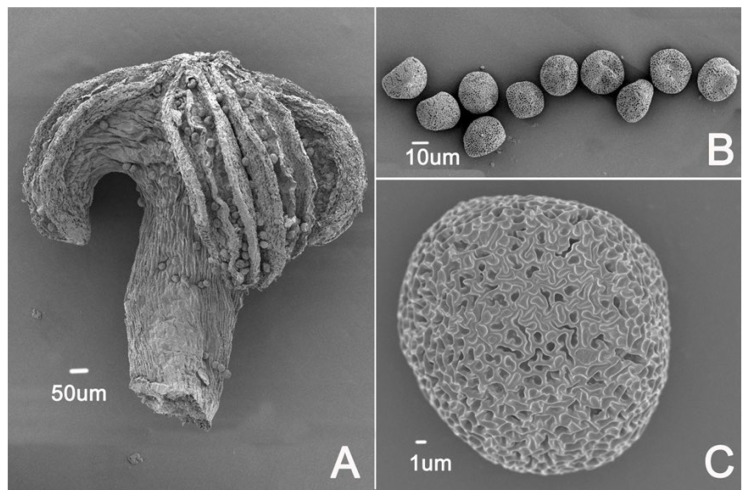
SEM micrograph of stamen and pollen morphology of *Cathetus yuanjiangensis* sp. nov. (voucher: Huan C. Wang et al. YJ31040, YUKU). (**A**) General view of the stamen, showing longitudinal dehiscence; (**B**) a group of scattered pollen grains; (**C**) close-up view of pollen grain, showing the reticulate exine ornamentation.

**Figure 5:**
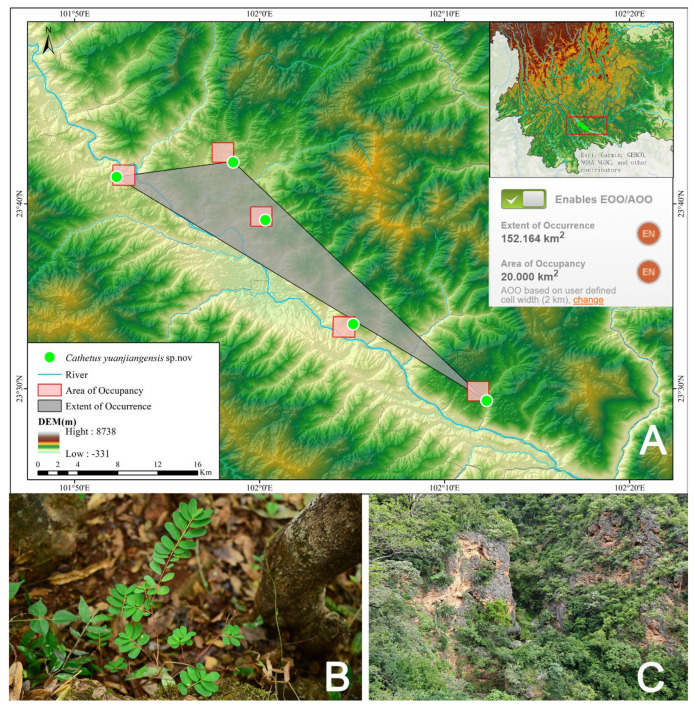
Distribution and habitat of *Cathetus yuanjiangensis.* (**A**) GeoCAT distribution map of *Cathetus yuanjiangensis* and estimation of extent of occurrence (EOO) and area of occupancy (AOO); (**B**) plant (voucher: Huan C. Wang YJ32404, YUKU); (**C**) habitat of the population found in Wadie.

*Cathetus yuanjiangensis* Huan C. Wang, Feng Yang et Chao Chen sp. nov. (Figure 3, Figure 4 and Figure 5).

Type: China. Yunnan Province: Yuxi City, near Yuanjiang County, Mount E’ka, 23°39′7.4″ N, 102°0′19.1″ E, 731 m, 6 September 2025, fl. and fr., Huan C. Wang et al. YJ31040 (Holotype: YUKU02074913!; Isotypes: KUN!, PE!, YUKU02074914!, YUKU02074915!).

Diagnosis: Morphologically, *Cathetus yuanjiangensis* is most similar to *C. beillei*, but differs clearly from the latter by being a small shrub, up to 50 cm high (vs. shrub, up to 3 m high in *C. beillei*), branchlets glabrous (vs. sparsely papillose-puberulent), stipules caducous (vs. more or less persistent), styles ca. 1.6 (vs. 0.5–0.8) mm long, connate into a ca. 5 (vs. 0.7) mm long column at base, stigmas spreading, slender, oblong, each bifid to 1/2 (vs. fleshy, triangular in outline, apex shallowly or deeply lobed, ca. 1 mm long).

Description: Evergreen small shrub, up to 50 cm tall, glabrous throughout. Stems several from a woody rhizome, much branched, branching non-phyllanthoid, 10–50 cm long, 1–2 mm in diam., purplish-red, reddish-brown, or grey-green when fresh, grey or greyish brown when old, bark peeling longitudinally into narrow strips. Leaves distichous, alternate, coriaceous, obovate-oblong or spatulate, 1.5–4.5 cm long, 1–2 cm wide, L/W ratio 1.5–2.25, on the lower parts of branchlets smaller, apex round, rarely retuse, base attenuate, cuneate to cordate, margin entire, slightly revolute when dry, adaxially green, abaxially greyish green; venation reticulate, conspicuous on both surfaces, lateral veins 15–20 pairs, midvein conspicuous abaxially; petiole short and stout, 1–2 mm long; stipules reddish-brown, ovate-triangular, membranous, ca. 2 mm long, base enlarged and auriculate, apex subulate to filiform, margin fimbriate or laciniate, caducous. Dioecious; flowers 1–3 per axil, borne on cushion-like swellings, base of swellings with numerous bracts; bracts similar to stipules, membranous, reddish-brown, deeply lacerate-fimbriate at base, persistent. Staminate flowers: 1–2 clustered; pedicel 3–5 mm long; sepals spreading, pale yellow, subhyaline, 6, 2-seriate, unequal, inner slightly longer than outer, outer 3 ovate-rhombic, inner 3 oblong, 1.5–2.0 mm long, 1.0–1.5 mm wide, margin membranous, nearly white, entire or lacerate, base thickened; stamens 3; filaments connate into a column, 2–3 mm long; anthers 3, ca. 0.6 mm long, perpendicular to filament column, longitudinally dehiscent; disc glands 6, on the inner sepals, pale yellow, semi-transparent. Pistillate flowers: 1–3 clustered; pedicel 5–7 mm long; sepals 6, slightly spreading, spaced evenly, 2-seriate, unequal, ovate-rhombic, 1.5–2.0 mm long, 1.0–1.5 mm wide, dark green or yellow-green, margin membranous, white, entire, base thickened, apex obtuse, distinct reticulate veins on sepals; styles 3, ca. 1.6 mm long, connate into a ca. 0.5 mm long column at base, stigmas spreading, slender, oblong, each bifid to 1/2, the lobes linear, recurved, spreading at less than 90°, brown in fruit stage; disc annular, encircling ovary; ovary globose, ca. 1 mm in diam., 3-loculed. Capsule subglobose, trigonous, ca. 6 mm in diam., ca. 4 mm thick, persistent sepals reflexed, apex umbilicate, with remnant black styles; pericarp thin, scabrous, with vermiform raised sculpturing, dark green or olive-green, glossy; surface with 3 ridges, depressed between ridges dividing into 3 carpels, dehiscing at maturity and separating from woody endocarp; each carpel 2-loculed, 1 seed per locule. Seeds light yellowish-brown or dark castaneous, trigonous-compressed globose or triangular-reniform, ca. 3 × 3 × 3 mm; testa thin, cells with prominent dark, domed centers.

Pollen morphology: The pollen grains of *Cathetus yuanjiangensis* are irregularly spherical, 25.08–28.77 × 21.74–26.90 µm, pantoporate, with a coarsely reticulate pattern on the surface (Figure 4). Ectoapertures usually irregular, slightly elongated pori, in shape resembling the lumina of the reticulum.

Distribution, habitat and conservation assessment: *Cathetus yuanjiangensis* is a rarely and poorly collected species endemic to the central Yunnan Province in Southwest China. As of now, it has been discovered in five different sites, all situated in the dry and hot valleys of the Yuanjiang River. This species typically inhabits moist streamside habitats, forest understories and margins of seasonal rainforests, at elevations of 500–1000 m (Figure 5). It has a restricted geographic range, with an estimated extent of occurrence (EOO) of 152.64 km^2^ and an area of occupancy (AOO) of 20 km^2^ (Figure 5). Based on IUCN Red List Categories and Criteria, we recommend that this species be listed in the Vulnerable (VU) category.

Phenology: The flowering and fruiting period is from July to December.

Etymology: The specific epithet “*yuanjiangensis*” is derived from the type locality of the new species, i.e., Yuanjiang County, Yunnan Province, China and the Latin suffix *ensis*, indicating the place of origin or growth.

Chinese name: Yuán Jiāng Shān Gé Mù, ㄩㄢˊ ㄐㄧㄤ ㄕㄢ ㄍㄜˊ ㄇㄨˋ (元江山格木).

Additional specimen examined (paratypes): China. Yunnan Province: Yuxi City, Yuanjiang County, Wadie Township, 23°29′21.39″ N, 102°12′16.73″ E, 897.58 m, 6 September 2025, fl., Chao Chen YJ31054 (YUKU); Yuanjiang County, Xiaoganba, 23°42′14.99″ N, 101°58′34.78″ E, 935.93 m, 21 September 2025, fl., Huan C. Wang YJ32404 (YUKU); Yuanjiang County, Shidie village, 23°33′30″ N, 102°5′4″ E, 977 m, 7 March 2026, nutrition period, Huan C. Wang et al. YJ33172 (YUKU); Yuanjiang County, Mandanqian Mountain, in savanna, under the forest, 23°41′27.62″ N, 101°52′17.15″ E, 740 m, 22 August 2012, fr, Yuanjiang County Census Team 5304281120 (IMDY0007449).

*Cathetus beillei* (Hutch.) Huan C. Wang et Feng Yang comb. nov.

≡ *Phyllanthus beillei* Hutch., D. Oliver et auct. suc. (eds.), Fl. Trop. Afr. 6(1): 733, 1912; *Phyllanthus welwitschianus* var. *beillei* (Hutch.) Radcl.-Sm., Kew Bull. 35: 775, 1980.

Types: Central African Republic. Source of the Ndelli River, 15–20 December 1902, Chevalier 6846 (lectotype: P, designated by Brunel and Roux [18]; isolectotype: K000406102!). Additional syntype: Central African Republic, Dar Banda, between Kaga Djé and the middle Bamingui, 5 December 1902, Chevalier 6612 (P, BR0000006243210!).

= *Phyllanthus stolzianus* Pax et Hoffm. ex Pax, Engl. Pflanzenw. Afr. 3(2): 27, 1921.

Types: Tanzanie. environs de Tukuyu (Kyimbila), 900 m, A. Stolz 1404 (Holotype: B, designated by Brunel and Roux [18]; Isotypes: C, HBG515876!, K, LD1403617, M0110580!, WAG0001374!).

= *Phyllanthus nyassae* Pax et K.Hoffm., Notizbl. Bot. Gart. Berlin-Dahlem 10: 383, 1928.

Types: Tanzanie. Nyassa-Hochland, Station Kyimbila, 900 m, A. Stolz 1891 (HBG515877!, JE00019679!, LD1403677!, M-0110581!).

= *Phyllanthus grahamii* Hutch. et M.B. Moss, Bull. Misc. Inform. Kew 1937: 413, 1937.

Types: Kenya. Arabuko, in forest undergrowth, 1927, R.M. Graham 1986 (Holotype: K, designated by Brunel and Roux [18]; Isotypes: Br, LISJC, P00539746!, PRE0487071-0!).

### 2.4. Key to Species of Cathetus Subgenus Cathetus

1a Leaf apex acute......................................................................................................................... 2
1b Leaf apex obtuse or mucronulate......................................................................................... 3
2a Young branches with a broad line of short crisped hairs along each side; staminate flowers with 4 sepals, 2 stamens, and 4 disk-glands............................ *Cathetus petraeus*
2b Branchlets completely glabrous; staminate flowers with 6 sepals, 3 stamens, and 6 disk-glands................................................................................................... *Cathetus gracilis*
3a Staminate flowers with 5 sepals and 5 disk-glands..................................... *Cathetus binhii*
3b Staminate flowers with 6 sepals and 6 disk-glands............................................................ 4
4a Plant a small shrub or more commonly a hemicryptophyte; leaf apex mucronulate................................................................................... *Cathetus welwitschianus*
4b Plant small shrub or more commonly shrubby; leaf apex rounded................................. 5
5a Leaves smaller, 1–2 cm long, 0.6–1.3 cm wide....................................... *Cathetus fasciculata*
5b Leaves larger, 1.5–7 cm long, 1–2 cm wide.......................................................................... 6
6a Stigma lobes thick and fleshy.......................................................................... *Cathetus beillei*
6b Stigma lobes distinctly spreading and slender.................................................................... 7
7a Leaf blade oblong-elliptic or narrowly obovate, 1.5–4 cm long, ca. 1 cm wide, L/W ratio 1.5–4; stigma lobes spreading at 180°, 0.1–0.2 mm long........... *Cathetus kerstingii*
7b Leaf blade oblong or spathulate, 1.5–4.5 cm long, 1–2 cm wide, L/W ratio 1.5–2.25; stigma lobes spreading at less than 90°, ca. 0.5 mm long......... *Cathetus yuanjiangensis*

## 3. Discussion

### 3.1. Generic Reassignment of Phyllanthus beillei Hutch

*Phyllanthus beillei* Hutch. was described by Hutchinson in 1912 based on two specimens collected from the Central African Republic [24]. Airy Shaw [25] noted that *P. stolzianus* Pax et K. Hoffm, *P. nyassae* Pax et K. Hoffm, and *P. grahamii* Hutch. et M.B. Moss should all be treated as synonyms of *P. beillei*. He further suggested that it was conspecific with a large-leaved variety of *P. fasciculatus* (Lour.) Muell.-Arg. (syn. *Cathetus fasciculata*) from northern Thailand and Cambodia, as cited by Beille [26] in *Flore Générale de l’Indo-chine*. Additionally, he observed a close relationship between *P. beillei* and *P. welwitschianus* Muell. Arg. Following an extensive examination of specimens from these and related taxa, Airy Shaw [27] formally merged *P. beillei* and its synonyms into *P. welwitschianus*. However, this taxonomic revision has not gained broad acceptance. While Radcliffe-Smith [28] proposed downgrading *P. beillei* to a subspecies of *P. welwitschianus*, the majority of authors maintain its status as a distinct species [18,20,21,29,30]. Based on specimen examination, morphological observation, palynology, and scanning electron microscopy evidence, Brune and Roux [18] confirmed that *P. beillei* was clearly different from *P. welwitschianus* by the habit (hemicryptophyte vs. shrub), plant height (≤0.5 m vs. up to 3 m), leaf base (truncate to subcordate vs. broadly cuneate to rounded), lateral vein (6–14 pairs vs. 12–23 pairs), staminal column length (1.3–1.6 mm vs. 0.5–0.7 mm), stigma lobes (thick and slightly spreading vs. slender and distinctly reflexed), and capsule size (7.5–8.0 mm vs. 6.5–7.0 mm) (Table 1). In this study, relevant specimens and field photographs of *P. welwitschianus* and *P. beillei* were examined. The results showed that there are stable and significant diagnostic characters between the two taxa, so we agree with the viewpoint of Brune and Roux [18] and treat them as distinct species. Recently, Bouman et al. [5], dealing with the genus *Cathetus*, left out and did not transfer *P. beillei* to *Cathetus*. Therefore, we propose transferring it to the genus *Cathetus* via a new taxonomic combination: *Cathetus beillei* (Hutch.) Huan C. Wang et Feng Yang.

### 3.2. The Plastid Genome Sequence of Cathetus yuanjiangensis

The plastid genome data were included primarily to facilitate phylogenetic analyses and comparative genomic assessment. The newly assembled plastomes provided the *mat*K, *acc*D-*psa*I, and *trn*S-*trn*G sequences incorporated into the plastid phylogenetic dataset. Furthermore, this study is the first to report the plastid genome for the new species. The genome structure and gene repertoire of the newly assembled plastid genome of *Cathetus yuanjiangensis* exhibited high consistency with previously published plastid genomes from other representative taxa of Phyllanthaceae, especially the congeneric species *C. clarkei* [31]. Notably, the intron loss of the *atp*F gene was also observed in *C. yuanjiangensis*, which was shared with *C. clarkei* and most other Phyllanthaceae species. In addition, no major structural rearrangements, gene losses or large-scale inversions were detected in the plastid sequence of *C. yuanjiangensis* when compared with these reference genomes. These data therefore support phylogenetic marker recovery and provide genomic resources for future comparative work in *Cathetus*, but they are not treated here as independent diagnostic evidence for species delimitation.

### 3.3. Phylogeny of Cathetus and Position of C. yuanjiangensis

The sampled species of *Cathetus* were recovered as a well-supported monophyletic group and were divided into two major highly supported clades in both nuclear and plastid phylogenetic trees, broadly consistent with the results of previous studies [3,30,32]. In the taxonomic treatment by Bouman et al. [5], these two clades were formally named subgen. *Cathetus* and subgen. *Macraea*. Our phylogenetic analyses recovered *Cathetus* to two major clades. One major clade comprised only members of subgen. *Macraea*, whereas the other included all sampled species of subgen. *Cathetus*, which were recovered as a sister group to a lineage containing *C. clarkei* (currently placed in subgen. *Macraea*) and a species allied to *C. fasciculata*. The placement of *C. clarkei* outside the main *Macraea* lineage renders subgen. *Macraea*, as currently circumscribed, polyphyletic. Accordingly, the circumscription of subgen. *Macraea* requires further re-evaluation.

*Cathetus yuanjiangensis* was placed within the clades corresponding to subgen. *Cathetus*, a placement further supported by both morphological and palynological evidence. Within this subgenus, *C. yuanjiangensis* was recovered as sister to the Asian species *C. fasciculata*. It shows a more distant relationship with African species such as *C. beillei* and *C. kerstingii*. These results provide clear support for assigning *C. yuanjiangensis* to subgen. *Cathetus*. However, because molecular data are currently available for only a limited proportion of the species attributed to this subgenus, the inferred sister relationship between *C. yuanjiangensis* and *C. fasciculata* should be regarded as provisional rather than as evidence that *C. fasciculata* is necessarily its closest extant relative. Subgen. *Cathetus* is distributed throughout the Old World tropics [5], but the relationships and divergence history of its Asian and African lineages remain poorly resolved. Denser taxon sampling across the geographic range of the subgenus, particularly from the Malay Archipelago and Australia, together with explicit biogeographic analyses, will be needed to clarify the origin and dispersal history of these lineages.

### 3.4. Morphological Distinctiveness of Cathetus yuanjiangensis

According to Bouman et al. [5], *Cathetus yuanjiangensis* should be assigned to *C.* subgen. *Cathetus* due to its stamen filaments completely connate into a column. Prior to the present study, only one species of the subgenus *Cathetus* was recorded in China, namely *C. fasciculata.* Indeed, *C. yuanjiangensis* forms a strongly supported sister clade with *C. fasciculata* in both the phylogenetic tree constructed based on the chloroplast data matrix and the nuclear gene data matrix. Despite their close phylogenetic placement, *C. yuanjiangensis* is morphologically distinct from *C. fasciculata* and all of its synonyms, including *P. serissifolius* Wall. ex Müll.Arg., *P. cinerascens* Hook. et Arn. and *P. roeperianus* Wall. ex Müll.Arg. *Cathetus yuanjiangensis* has relatively larger leaves, 1.5–4.5 cm long, 1–2 cm wide, staminate flowers with spreading sepals, 1.5–2.0 mm long, filaments connate into a column, ca. 2–3 mm long, longer than the sepals, styles 3, ca. 1.6 mm long, connate into a ca. 5 mm long column at base, stigmas spreading, slender, oblong, each bifid to 1/2, the lobes linear. In contrast, *C. fasciculata* has smaller leaves, 1–2 cm long, 0.6–1.3 cm wide, staminate flowers sepals arranged in a campanulate form, ca. 1.3 mm long, filaments connate into a column, shorter than the sepals, styles ca. 1.1 mm long, connate at middle to base, bifid at apex, lobes oblong.

Practically, *Cathetus yuanjiangensis* is most similar to *C. beillei* in terms of morphology, which is widely distributed in Tropical Africa and Indo-China (Cambodia and Thailand) [18]. However, it differs clearly from *C. beillei* by being a small shrub, up to 50 cm high (vs. shrub, up to 3 m high in *C. beillei*), branchlets glabrous (vs. sparsely papillose-puberulent), stipules caducous (vs. more or less persistent), styles ca. 1.6 (vs. 0.5–0.8) mm long, connate into a ca. 5 (vs. 0.7) mm long column at base, stigmas spreading, slender, oblong, each bifid to 1/2 (vs. fleshy, triangular in outline, apex shallowly or deeply lobed, ca. 1 mm long). *Cathetus yuanjiangensis* is also morphologically similar to *C. kerstingii* (Brunel) R. W. Bouman from West Tropical Africa. *Cathetus yuanjiangensis* differs from *C. kerstingii* in having obovate-oblong to spatulate leaves (vs. oblong-elliptic or narrowly obovate-membranous), L/W ratio 1.5–2.25 (vs. 1.5–4.0), styles ca. 1.6 (vs. 0.2–0.5) mm long, stigma lobes ca. 0.5 (vs. 0.1–0.2) mm long, spreading at less than 90° (vs. at 180°). A qualitative characters comparison between *C. yuanjiangensis*, *C. fasciculata*, *C. beillei* and *C. kerstingii* is listed in Table 1.

Notably, the comparison also reveals a morphological-molecular incongruence. *Cathetus beillei* is the most similar species morphologically with *C. yuanjiangensis*, whereas *C. fasciculata* is the closest sampled relative in both nuclear and plastid trees. The similarity between *C. yuanjiangensis* and *C. beillei* is concentrated mainly in the shape and size of the leaf blade, which should probably be the result of convergent evolution, as they are harbored in the understory of forests in arid zones.

Pollen grains of *Cathetus yuanjiangensis* are irregularly spherical, measuring 25.08–28.77 × 21.74–26.90 µm. They are periporate with a coarse reticulate exine ornamentation, conforming to the “*Phyllanthus cochinchinensis* type” as defined by Punt [30]. This pollen morphology supports the placement of *C. yuanjiangensis* within *C.* subgen. *Cathetus* and is consistent with the pollen types of closely related taxa [18,26,33,34,35]. This congruence in pollen morphology demonstrates evolutionary stasis (or conservatism) of palynological traits within this lineage.

### 3.5. Conservation Assessment of Cathetus yuanjiangensis

Based on the assessment using the GeoCAT tool [36], the extent of occurrence (EOO) of *Cathetus yuanjiangensis* is 152.164 km^2^, and its area of occupancy (AOO) is only 20 km^2^. Fortunately, five known distribution sites are situated within the Yunnan Yuanjiang National Nature Reserve, ensuring that the wild population receives effective in situ protection. Based on the IUCN Red List criteria [37], it should be classified as a Vulnerable (VU) species. Furthermore, ex situ conservation efforts are underway; the Xishuangbanna Tropical Botanical Garden (XTBG) has successfully introduced and cultivated this species under introduction number 0020091695, and the Yuanjiang Savanna Ecosystem Research Station has also achieved successful introduction and cultivation. Leveraging a mature ex situ conservation system, XTBG provides a stable cultivation environment, effectively safeguarding the species against catastrophic extinction of wild populations due to stochastic events or human destruction. The Yuanjiang Savanna Ecosystem Research Station is located within the Yuanjiang River basin. Its climate and soil types are highly congruent with the native habitats of *C. yuanjiangensis*, facilitating better post-introduction adaptation and maximizing the preservation of the species’ innate adaptive traits. This establishes a solid foundation for future reintroduction. This dual-site introduction model has successfully established a double guarantee for the ex situ conservation of *C. yuanjiangensis*.

## 4. Materials and Methods

### 4.1. Morphological and Taxonomic Analyses

The morphological studies of the new species were conducted on living plants and specimens coming from the five localities corresponding to the type and paratypes. Digital images of this genus are available at online databases, including the iNaturalist (https://www.inaturalist.org), the Tropicos (www.tropicos.org), the JSTOR Global Plants (http://plants.jstor.org/), the Global Biodiversity Information Facility (GBIF, https://www.gbif.org/, accessed on 20 July 2026), and the Chinese Virtual Herbarium (CVH, https://www.cvh.ac.cn/), as well as collections housed at CDBI, IBSC, KUN, PE and YUKU; these were extensively examined and compared with the new species. Pertinent taxonomic literature was consulted [5,16,17,18,19,20,21,22,23].

Morphological data for *Cathetus kerstingii* (J.F.Brunel) R.W.Bouman [18], *C. binhii* (Thin) R.W.Bouman [19], and *C. petraeus* (A.Chev. et Beille) R.W.Bouman [38] were extracted from their respective protologues. Morphological characters for *C. gracilis* (Hassk.) R.W.Bouman were extracted from the descriptive treatment of *Phyllanthus albidiscus* (Ridl.) Airy Shaw provided by van Welzen and Chayamarit [22]. Descriptions of *C. welwitschianus* (Müll.Arg.) R.W.Bouman and *C. beillei* follow Brune and Roux’s revision [18]. We adopt the species circumscription of *C. fasciculata* proposed in the taxonomic revision by Chakrabarty and Balakrishnan [39,40], which excludes *Phyllanthus roeperianus* Wall. ex Müll.Arg. Morphological data for this taxon are retrieved from Flora Reipublicae Popularis Sinicae [17].

### 4.2. Palynological Studies

For palynological comparison with morphologically similar species, the pollen grains of *Cathetus yuanjiangensis* were observed under a scanning electron microscope. Mature pollen grains of the new species were sampled from the type materials and attached directly to carbon adhesive tape. They were coated with gold-palladium using a BAL-TEC SCD 005 cool sputter coater (BAL-TEC AG, Balzers, Liechtenstein) at Yunnan University, Kunming, China. Observations were conducted using a Regulus 8100 scanning electron microscope (SEM) (Hitachi, Tokyo, Japan) at 3.0 KV at a magnification of 3.00 K×. Descriptive terminology for the pollen grains follows Punt et al. [30].

### 4.3. Conservation Assessment

Conservation status was assessed using GeoCAT (online tool available at https://geocat.iucnredlist.org/) [36] to estimate the extent of occurrence (EOO) and the area of occupancy (AOO) of the species, followed by applying the IUCN Red List Categories and Criteria [37] for conservation status assessment.

### 4.4. Molecular Analysis

#### 4.4.1. Sample Collection, DNA Extraction and Sequencing

Fresh leaves of *Cathetus yuanjiangensis* were collected from living plants in Yuanjiang County, and voucher specimens (Collection Number: Huan C. Wang et al. YJ31040 and Chao Chen YJ31054) were deposited at YUKU. The genomic DNA was extracted from fresh leaves. The short-insertion library (350 bp) was constructed and then sequenced to obtain 2 × 150 bp paired-end data by using the Illumina NovaSeq 6000 platform (Illumina, San Diego, CA, USA) at Novogene (Beijing, China), and 10 GB of raw data was obtained.

#### 4.4.2. Plastid Genome Assembly and Annotation

The raw data for the new species were filtered through Trimmomatic v. 0.39 [41] to obtain a clean dataset. The plastid genomes were assembled using GetOrganelle v1.7.7.1 [42] with default parameters. Initially, the online program GeSeq with default parameters [43] was utilized to annotate the complete plastome. The putative start and stop codons of protein-coding genes were manually adjusted in Geneious Prime 2020.0.3, using the published plastome of *Cathetus clarkei* as the reference [31]. The resulting circular physical map of the plastome of *C. yuanjiangensis* was drawn using CPGview [44].

#### 4.4.3. Nuclear Data Extraction

Whole nuclear ribosomal DNA repeat unit (nrDNA) comprising the complete sequence of 18S–5.8S–26S genes, as well as non-coding external and internal transcribed spacers (ETS and ITS), were assembled using the GetOrganelle v1.7.7.1 [42] with default parameters. The phytochrome C (*PHYC*) gene of *Cathetus fasciculata* (GenBank accession number: MN904262) was used as the reference genome for the *PHYC* assembly. We used BWA v. 0.7.17 with default parameters [45] to facilitate the reads mapping to the reference gene. SAMtools v.0.1.18 with default settings [46] converts the SAM file to BAM format while filtering out unmapped sequences and low-quality sequences. Paired mapped sequences are then extracted from the filtered BAM file and output as paired-end fastq files, with unpaired sequences and single-end sequences discarded. The extracted data were assembled into contigs using SPAdes [47]. A BLAST database is constructed from the assembly results using NCBI BLAST+ (v2.16.0), followed by blastn alignment to screen for the best-matching contig. Finally, the sequence file with the highest homology to the reference sequence is obtained.

#### 4.4.4. Dataset and Phylogenetic Analysis

We utilize two nuclear (high-copy spacer ITS and low-copy *PHYC*) and three plastid (*acc*D-*psa*I, *trn*S-*trn*G and *mat*K) DNA markers and constructed phylogenetic trees using two sets of matrices with the Maximum Likelihood (ML) and Bayesian Inference (BI) methods. The three plastid DNA markers of *Cathetus fasciculata* and *C. clarkei* were extracted from the plastid genome; the ITS sequences of *C. fasciculata* were extracted from whole nrDNA; the rest were downloaded directly from GenBank (Table A2).

The sequences were aligned using MAFFT v.7.490 [48] and trimmed using trimAl v.1.4.1 [49], in which poorly aligned regions were removed. Then, the ITS region was concatenated with *PHYC*, while *acc*D-*psa*I, *trn*S-*trn*G, and *mat*K were concatenated together, resulting in the construction of two datasets (nuclear and plastid).

Taxon sampling in this study was limited by the availability of comparable molecular data. Although approximately 40 species have been described in *Cathetus*, only a subset is currently represented by multilocus sequences appropriate for the phylogenetic matrices used here. Consequently, the concatenated nuclear dataset included 30 sequences of *Cathetus*, and the concatenated plastid regions included 28 sequences. Nevertheless, our sampling covers both subgenera of *Cathetus*. Under these circumstances, our analyses were performed to assess the phylogenetic position of *C. yuanjiangensis* among the sampled taxa. For the phylogenetic analysis, we selected outgroups based on the phylogenetic tree of *Phyllanthus* sensu lato established by Bouman et al. [5], using *Nymphanthus sootepensis* (Craib) R.W.Bouman and *Kirganelia glauca* (Wall. ex Müll. Arg.) R.W.Bouman as outgroups.

For the concatenated nuclear dataset and plastid regions, the best-fit substitution models were identified via the Bayesian Information Criterion (BIC) using ModelFinder [50]. The maximum-likelihood tree was inferred using IQ-TREE v.1.6.12 [51], and the ultrafast bootstrap feature “-bb 1000” [52] was used to obtain branch support. The BI analysis was performed using MrBayes 3.2.2 [53], with evolutionary rate heterogeneity modeled by 4 gamma categories; two parallel runs were performed, each comprising 4 chains (1 heated and 3 cold) for 100,000,000 generations, with sampling carried out every 1000 generations. The first 25% of samples were discarded as burn-in. All the phylogenetic trees were visualized in FigTree v.1.4.4 and edited in WPS Office 12.1.0.26899.

## Figures and Tables

**Table 1:** Qualitative character comparison among *Cathetus yuanjiangensis*, *C. fasciculata*, *C. welwitschianus*, *C. beillei* and *C. kerstingii*.

Characters		Species				
		*C. yuanjiangensis*	*C. fasciculata*	*C. welwitschianus*	*C. beillei*	*C. kerstingii*
**Habit**		Evergreen small shrub, up to 50 cm tall	Shrub, up to 3 m tall	Erect perennial herb or subshrub, up to 90 cm tall	Small much-branched shrub, up to 3 m tall	Shrubby, up to 1.5 m tall
**Indumentum**		Glabrous throughout	Young branchlets yellowish brown pubescent, glabrescent with age; petioles velvety-papillate	Glabrous or finely pubescent	Branchlets sparsely papillose-puberulent, glabrescent elsewhere; petioles papillose-puberulent	Young branchlet apices pubescent, bark peeling longitudinally in narrow strips with age, branchlets glabrous
**Leaf**	**Morphology**	Coriaceous, obovate-oblong to spatulate, 1.5–4.5 cm long, 1–2 cm wide, L/W ratio 1.5–2.25; apex obtuse-rounded, rarely retuse; base attenuate, cuneate to cordate	Coriaceous, obovate, oblong-obovate or spathulate, 1–2 cm long, 0.6–1.3 cm wide; apex obtuse-rounded, very rarely retuse; base attenuate	Subcoriaceous, suborbicular to elliptic-oblong, 0.5–3.5 cm long, 0.3–2.8 cm wide; apex rounded to retuse, sometimes mucronulate; base rounded, truncate and subcordate	Subcoriaceous, oblong-elliptic or narrowly obovate, 2–5 cm long, 1–2 cm wide; apex rounded; base broadly cuneate	Membranous, oblong-elliptic to narrowly obovate, 18–25 mm long, 7–15 mm wide; apex rounded; base rounded to broadly cuneate
	**Venation**	Reticulate veins distinct on both surfaces, lateral veins 15–20 pairs, midrib more prominent abaxially	Midrib slightly prominent on both surfaces, lateral veins obscure	Lateral veins 6–18 pairs, nearly obscure or slightly distinct adaxially, more distinct abaxially	Lateral veins ca. 11 pairs per side, slightly prominent, spreading, reticulate	Lateral veins 12–20 pairs, slightly prominent abaxially, reticulate
**Stipule**		Reddish brown, membranous, ovate-triangular, base enlarged and auriculate, apex subulate to filiform, ca. 2 mm long, margin fimbriate or laciniate, caducous	Reddish brown, ovate-triangular, ca. 2 mm long, base cordate, margin ciliate, apex caudate-acuminate, persistent	-	More or less persistent, subulate-triangular, 4 mm long, 1 mm wide at base, apex filiform, usually brown-ciliate	Scarious, narrow-triangular, base auriculate, 1.3–3.7 mm long, 0.9–1.0 mm wide at insertion, persistent
**Staminate flower**	**Sepal**	6, spreading, 2-seriate and unequal; outer ovate-rhombic, inner oblong, 1.5–2.0 mm long, 1–1.5 mm wide, margin membranous and subwhite, base thickened	6, obovate to spathulate, ca. 1.3 mm long, 1–1.2 mm wide, base thickened, margin membranous	6, white to yellowish green with red	6, 2-seriate, broadly elliptic, ca. 3 mm long, apex rounded to truncate, glabrous	6,2-seriate, dimorphic; outer membranous, 0.8–1.1 mm long; inner fleshy, apex truncate, 0.7–1.1 mm long, all 0.6–1.0 mm wide
	**Stamen**	3, filaments connate into a column 2–3 mm long; anthers 3, ca. 0.6 mm long, perpendicular to filament column, longitudinally dehiscent	3, filaments connate to the middle; thecae parallel, longitudinally dehiscent		3, filaments connate into a column 2 mm long	3, filaments connate into a column 0.4–0.5 mm long; anthers pendulous, 0.4–0.5 mm long, apices connate
**Pistillate flower**	**Sepal**	6, slightly spreading, 2-seriate, ovate-rhombic, 1.5–2.0 mm long, 1.0–1.5 mm wide, dark green or yellowish green, margin membranous and white, base thickened, with distinct reticulate veins	6, ovate or ovate-rhombic, 1.5–1.8 mm long, ca. 1.5 mm wide, margin membranous, base thickened	6, white to yellowish green with red, size not mentioned	6, spreading, 2-seriate, broadly elliptic or suborbicular, 3 mm long, apex rounded or subtruncate, glabrous, with distinct venation	6, dimorphic; outer 1.6–2.0 mm long, 1.3–1.5 mm wide; inner 1.8–2.0 mm long, 1.7–1.9 mm wide, with branched midrib
	**Style**	3, ca. 1.6 mm long; lower part connate into a column 0.5 mm long, stigmas spreading, slender, oblong, each bifid to 1/2, lobes linear, spreading at less than 90°	3, ca. 1.1 mm long, connate from middle to base, apex bilobed and recurved	-	3, connate into a column 0.7 mm long; stigmas 3, spreading and fleshy, triangular in outline, apex lobed, lobes ca. 1 mm long, thick and fleshy	3, basal part connate into a column 0.1–0.3 mm long; lobes spreading at 180°, slender and cylindrical, 0.1–0.2 mm long, brown in fruit stage

## Data Availability

The original contributions presented in this study are included in the article. Further inquiries can be directed to the corresponding authors.

## Appendix A

**Table A1:** Scientific names, voucher information, and GenBank accession numbers (ITS, *PHYC*, *acc*D-*psa*I, *trn*S-*trn*G and *mat*K) of the specimens used in this study. A dash (–) indicates missing data.

ID	Voucher	ITS	*PHYC*	*acc*D-p*sa*I	*trn*S-*trn*G	*mat*K
*C. beckleri*	Australia, Hosking 2680 (NE)	MN915861	MN904231	MN915347	MN915618	MN916127
*C. chrysanthus*	New Caledonia, Munzinger and McPherson 796 (MO)	AY936680	–	–	–	AY936585
*C. distichus*	USA, Hawai’i, Harold st. John 17.985 (L)	MN915912	MN904276	MN915404	MN915665	MN916163
*C. exilis*	Australia, Hunter et al. 1528 (L)	MN915922	MN904283	–	MN915672	MN916362
*C.* aff. *fasciculata* 1	China, Yunnan, Xishuangbanna Tropical Botanical Garden, Bouman and Yong RWB052 (HITBC)	MN915840	MN904250	MN915324	MN915601	MN916144
*C.* aff. *fasciculata* 2	China, Yunnan, Xishuangbanna Tropical Botanical Garden, Bouman and Yong RWB065 (HITBC)	MN915841	MN904251	MN915325	MN915602	MN916145
*C.* aff. *fasciculata* 3	China, Yunnan, Xishuangbanna Tropical Botanical Garden, Bouman and Yong RWB060 (HITBC)	MN915842	MN904252	MN915326	MN915603	MN916146
*C. fasciculata* 1	China, Xia et al. s.n. (K)	AY936684	–	–	–	AY936589
*C. fasciculata* 2	China, Hong Kong, Bouman et al. RWB026 (L)	MN915895	MN904262	MN915384	MN915648	MN916154
*C. filicaulis*	Australia, Telford 13516 (NE)	MN915923	MN904284	MN915415	MN915673	MN916170
*C. gardnerianus*	Sri Lanka, Kathriarachchi et al. 42 (K)	AY936694	MN904314	MN915429	MN915684	AY936598
*C. glaucophyllus* 1	Guinea, Van der Brugt 1156 (WAG)	MN915938	MN904317	MN915432	MN915687	MN916183
*C. glaucophyllus* 2	Guinea, Haba 123 (WAG)	MN915939	MN904318	MN915433	MN915688	MN916340
*C. kerstingii* 1	Guinea, Malaisse 14792 (WAG)	MN915951	–	MN915448	MN915702	–
*C. kerstingii* 2	Guinea, Darbyshire 562 (WAG)	MN915950	MN905074	MN915447	MN915701	MN916189
*C. myrtifolius* 1	Sri Lanka; Kathriarachchi et al. 12 (K)	AY936712	–	–	–	AY936616
*C. myrtifolius* 2	China, Hong Kong University campus, Bouman and Liu RWB034 (L)	MN915995	MN904370	MN915495	MN915736	MN916214
*C. myrtifolius* 3	China, Yunnan, Xishuangbanna Tropical Botanical Garden, Bouman and Yong RWB053 (HITBC)	MN915996	MN904371	MN915496	MN915737	MN916215
*C. myrtifolius* 4	Singapore, Singapore Botanical Garden, Yu 58 (L)	MN915997	MN904372	–	MN915738	MN916216
*C. petraeus*	Liberia, Blyden 1037 (WAG)	MN916026	MN904397	MN915524	MN915763	MN916239
*C. ussuriensis*	Japan, Kawakita 124 (KYO)	–	FJ235366	–	–	FJ235274
*C. virgatus* 1	Australia, Wrigley and Telford 46642 (K)	AY936738	MN904442	MN915574	MN915807	AY936639
*C. virgatus* 2	China, Hong Kong, Bouman RWB069 (L)	MN916074	MN904440	MN915572	MN915805	MN916276
*C. virgatus* 3	China, Hong Kong, Bouman RWB070 (L)	MN916075	MN904441	MN915573	MN915806	MN916277
*C. wheeleri*	Sri Lanka, Kathriarachchi et al. 33 (K)	AY936740	MN904445	MN915577	MN915810	AY936641
*C. beillei* 1	Tanzania; Bidgood et al. 1882 (K)	AY936739	–	–	–	AY936640
*C. beillei* 2	Thailand, JM-40231	AB550103	–	–	–	–
*C. clarkei* 1	China, Yunnan, S. X. Luo 425 (IBSC)	HM106989	–	–	–	–
*C. clarkei* 2	China, Yunnan, Min Fan FM2022111811	–	–	extracted from the plastid genome, OR683690		
*C. yuanjiangensis* 1	China, Yunnan, Wang HuanChong et al. YJ31040 (YUKU)	PZ230145	PZ225068	extracted from the assembled plastid genome, PZ210709		
*C. yuanjiangensis* 2	China, Yunnan, Yuanjiang, Chen Chao YJ31054 (YUKU)	PZ230144	PZ225069	extracted from the assembled plastid genome, PZ210710		
*Nymphanthus sootepensis*	Myanmar, Makino banical garden expedition (2015) 103753 (MBK)	MN916060	MN904427	MN915559	MN915791	MN916266
*Kirganelia glauca*	China, Hong Kong, Bouman and Liu RWB028 (L)	MN915940	MN904291	MN915434	MN915689	MN916175

**Table A2:** List of genes was annotated in the chloroplast genome of Cathetus yuanjiangensis.

Category	Gene Group	Gene Name	Number
**Photosynthesis**	Subunits of photosystem I	*psa*B, *psa*A, *psa*I, *psa*J, *psa*C	5
	Subunits of photosystem II	*psb*A, *psb*K, *psb*I, *psb*M, *psb*D, *psb*C, *psb*Z, *psb*J, *psb*L, *psb*F, *psb*E, *psb*B, *psb*T, *psb*N, *psb*H	15
	Subunits of NADH dehydrogenase	*ndh*J, *ndh*K, *ndh*C, *ndh*B(2) #, *ndh*F, *ndh*D, *ndh*E, *ndh*G, *ndh*I, *ndh*A #, *ndh*H	12
	Subunits of cytochrome b/f complex	*pet*N, *pet*A, *pet*L, *pet*G, *pet*B #, *pet*D #	6
	Large subunit of rubisco	*rbc*L	1
	Subunits of ATP synthase	*atp*A, *atp*F, *atp*H, *atp*I, *atp*E, *atp*B	6
**Self-replication**	Proteins of large ribosomal subunit	*rpl*33, *rpl*20, *rpl*36, *rpl*14, *rpl*16 #, *rpl*22, *rpl*2(2) #, *rpl*23(2), *rpl*32	11
	Proteins of small ribosomal subunit	*rps*2, *rps*14, *rps*4, *rps*18, *rps*12(2) ##, *rps*11, *rps*8, *rps*3, *rps*19, *rps*7(2), *rps*15	13
	Subunits of RNA polymerase	*rpo*C2, *rpo*C1 #, *rpo*B, *rpo*A	4
	Ribosomal RNAs	*rrn*16S(2), *rrn*23S(2), *rrn*4.5S(2), *rrn*5S(2)	8
	Transfer RNAs	*trn*K-UUU #, *trn*Q-UUG, *trn*S-GCU, *trn*S-CGA #, *trn*R-UCU, *trn*C-GCA, *trn*D-GUC, trnY-GUA, *trn*E-UUC(3) #, *trn*T-GGU, *trn*S-UGA, *trn*G-GCC, *trn*M-CAU(4), *trn*S-GGA, *trn*T-UGU, *trn*L-UAA #, *trn*F-GAA, *trn*V-UAC #, *trn*W-CCA, *trn*P-UGG, *trn*L-CAA(2), *trn*V-GAC(2), *trn*A-UGC(2) #, *trn*R-ACG(2), *trn*N-GUU(2), *trn*L-UAG	36
**Other genes**	Maturase	*mat*K	1
	Protease	*clp*P ##	1
	Envelope membrane protein	*cem*A	1
	Acetyl-CoA carboxylase	*acc*D	1
	c-type cytochrome synthesis gene	*ccs*A	1
	Conserved open reading frames	*ycf*3 ##, *ycf*4, *ycf*2(2), *ycf*1(2)	6
**Total**			128

#: one intron number, ##: two intron number, (n): gene copy number.
